# Mapping the clinical trial landscape of multiple primary lung cancer in the era of precision oncology: persistent exclusion and design limitations hamper evidence-based treatment development

**DOI:** 10.3389/fonc.2025.1642062

**Published:** 2025-08-04

**Authors:** Jianwei Shi, Linchuan Liang, Yushun Gao

**Affiliations:** ^1^ Department of Thoracic Surgery, National Cancer Centre/National Clinical Research Centre for Cancer/Cancer Hospital, Chinese Academy of Medical Sciences and Peking Union Medical College, Beijing, China; ^2^ Nuffield Department of Primary Care Health Sciences, University of Oxford, Oxford, United Kingdom

**Keywords:** multiple primary lung cancer (MPLC), clinical trial design, patient underrepresentation, precision oncology, tumor heterogeneity

## Abstract

Multiple primary lung cancer (MPLC) is increasingly recognized for its clinical and biological significance, yet it remains strikingly underrepresented in clinical trials. We systematically analyzed 8,212 lung cancer trials registered between 2015 and 2024 across four major international registries, finding that only 18 (0.22%) explicitly included MPLC patients. Most of these trials were early-phase, observational, and surgery-focused, with minimal incorporation of biomarker-driven or precision strategies. This underrepresentation reflects a structural exclusion rooted in traditional single-lesion trial paradigms. It is further exacerbated by limited engagement from industry and government sponsors due to high trial complexity and low commercial incentive. Inclusion-oriented frameworks are urgently needed to align research with MPLC’s clinical reality.

## The clinical invisibility of MPLC

Multiple primary lung cancer (MPLC), defined as two or more anatomically distinct and histologically confirmed primary tumors occurring synchronously or metachronously within the lung, is increasingly recognized due to the widespread use of high-resolution imaging and refined histopathologic criteria, yet it remains largely overlooked in clinical research. MPLC is a unique clinical entity characterized by lesions at various histologic stages—from adenocarcinoma *in situ* (AIS) to invasive adenocarcinoma (IAC)—and by distinct molecular profiles (1).

A growing concern is the persistent exclusion of MPLC patients from prospective clinical trials, particularly those evaluating systemic therapies or biomarker-driven interventions. Trial eligibility criteria frequently assume single-lesion homogeneity and uniform staging, thereby disqualifying patients with multifocal or temporally separated tumors. This exclusion is further entrenched by conventional staging systems such as the TNM classification (2), which is inherently designed for single-lesion evaluation and fails to accommodate clonally independent tumor foci. As a result, the applicability of evidence-based guidelines to MPLC patients is limited, creating a gap between research frameworks and clinical reality (3).

In light of these challenges, it is critical to examine how MPLC is represented—or excluded—across current clinical trials and to identify the structural barriers embedded in trial design that restrict the generation of applicable evidence.

## Trial registry analysis across global platforms

To investigate the representation of MPLC in ongoing clinical research, we systematically reviewed interventional trials related to lung cancer registered between 2015 and 2024 across four major trial registries: ClinicalTrials.gov (4) (United States), the Chinese Clinical Trial Registry (5) (ChiCTR), the EU Clinical Trials Register (6) (EU-CTR), and the International Standard Randomized Controlled Trial Number (7) (ISRCTN) registry. These databases collectively cover a broad spectrum of industry-sponsored and investigator-initiated studies across North America, Europe, and Asia.

We searched for trials containing keywords (Boolean syntax, applied to “Other terms/Keywords” field in each registry): “multiple primary lung cancer” OR “multiple primary lung cancers” OR “multifocal lung cancer” OR “multiple primary” OR “synchronous lung cancer” OR “metachronous lung cancer” OR “MPLC” within the eligibility criteria, study objectives, or summaries. Studies were included if they explicitly mentioned MPLC, involved patients with pathologically distinct multifocal lesions, or addressed therapeutic, diagnostic, or prognostic strategies relevant to this population. Full registry export files (XML/CSV) were downloaded on May 2025.

Screening and eligibility. Two authors (J.S., Y.L.) independently screened titles/abstracts and full records. Agreement was excellent (Cohen’s κ = 0.89); discrepancies (6%) were resolved by discussion. Trials were included if they (i) explicitly mentioned MPLC or a synonym, (ii) enrolled patients with pathologically or radiologically distinct multifocal lung lesions, and (iii) evaluated a therapeutic, diagnostic, or prognostic intervention.

Studies focusing solely on metastatic recurrence, purely observational cohorts, withdrawn/terminated entries with no results, or duplicate records across registries were excluded. The screening process is depicted in **Supplementary Figure S1** (PRISMA diagram).

Eligible trials were further categorized by intervention type (e.g., surgery, systemic therapy, or combined modalities), trial phase, and sponsor classification (industry vs. academic). Annual trial frequencies were calculated to visualize temporal trends, and proportions were assessed to determine research focus areas. This registry-based mapping provides a comprehensive overview of how MPLC is currently positioned within the global landscape of lung cancer trials and reveals structural gaps in both patient inclusion and trial design.

Building on this comprehensive screening workflow, we next report the quantitative mapping of MPLC representation across global trial registries.

## Quantifying structural underrepresentation

To assess the representation of MPLC in clinical research, we systematically analyzed prospective lung cancer trials registered between 2015 and 2024 across ClinicalTrials.gov, ChiCTR, EU-CTR, and ISRCTN. Of the 8,212 lung cancer trials identified after de-duplication, only 18 (0.22%) explicitly referenced MPLC in their eligibility criteria or objectives. Although the global volume of lung cancer clinical research has expanded rapidly over the past decade—rising from fewer than 1,000 trials in 2015 to over 8,000 by 2024—MPLC-specific studies have remained exceedingly rare. This persistent underrepresentation underscores a structural eligibility gap.

**Figure 1:**
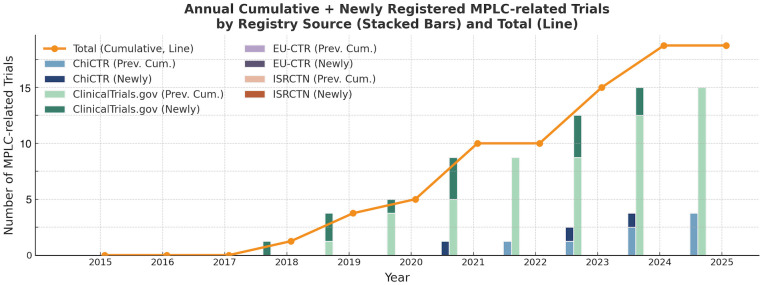
Annual cumulative and newly registered MPLC-related trials by registry source (2015–2024).

To dissect this underrepresentation, we examined registry-specific contributions (**Figure 1**). ClinicalTrials.gov accounts for the majority of MPLC-related registrations, while ChiCTR exhibits a recent but limited increase. In contrast, EU-CTR and ISRCTN contributed few MPLC trials over the examined period. Despite a slow upward trend, the cumulative number remains exceedingly low, underscoring the systemic exclusion of MPLC patients from prospective clinical investigation.

Each bar represents the number of MPLC-related trials newly registered in a given year, overlaid upon prior cumulative totals. The orange line traces the annual cumulative total of all MPLC-related trials.

Among the 18 studies explicitly referencing MPLC, the majority were observational, a tendency that favors descriptive or registry-based investigations rather than interventional design (8, 9). Furthermore, an intervention analysis revealed that nearly half of these trials lacked explicit treatment strategies in their titles. Where specified, surgery was the dominant approach, with only limited engagement with immunotherapy, targeted therapy, or combination protocols, reflecting a broader scarcity of molecularly guided approaches tailored to multifocal disease (10–12). The limited incorporation of biomarker stratification and combined-modality protocols indicates a research framework largely misaligned with the biological complexity of MPLC, underscoring the conceptual and infrastructural lag in trial design tailored to MPLC’s unique biology (13).

**Figure 2:**
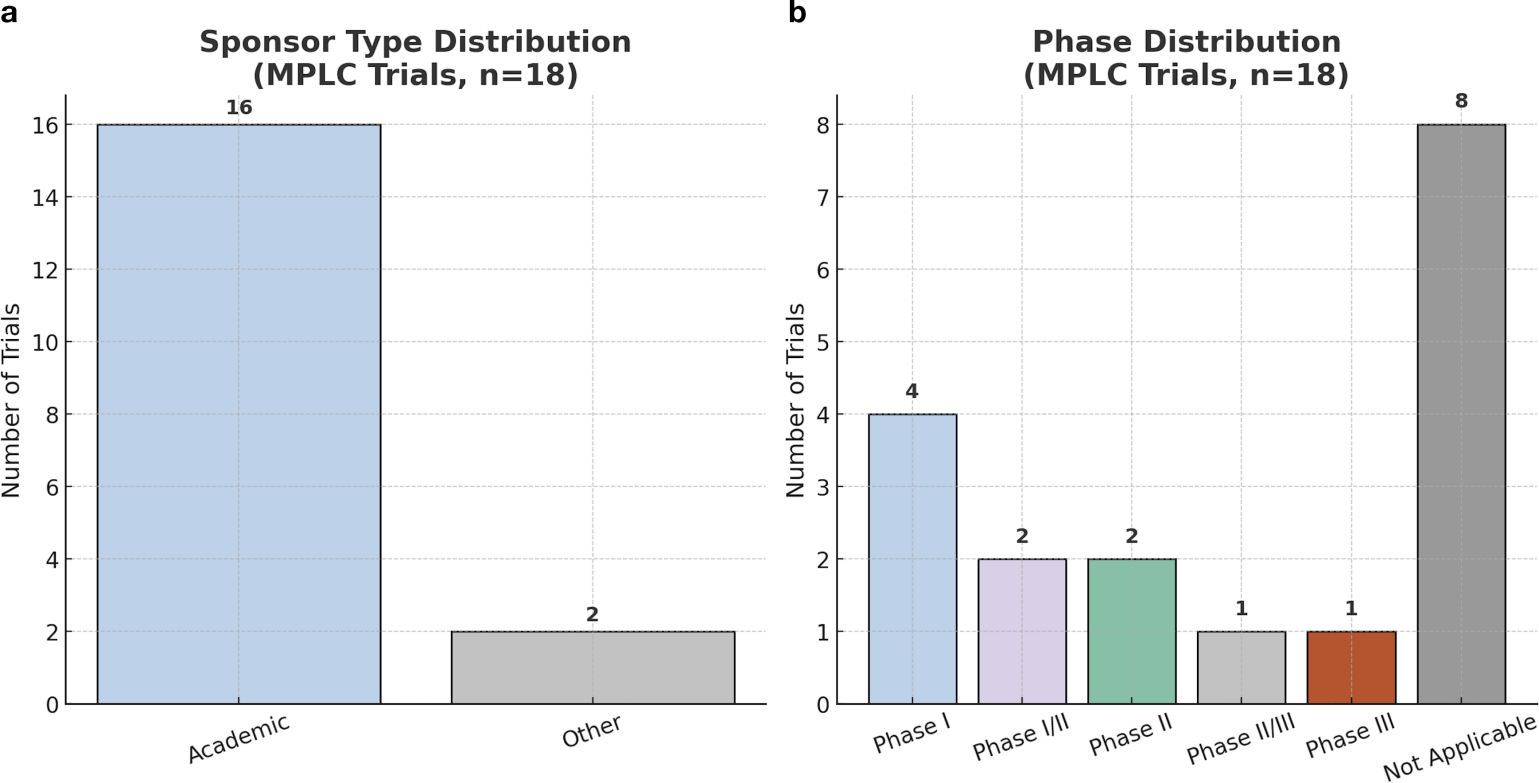
Sponsor type and phase classification of MPLC-related clinical trials. **(a)** Sponsor-type distribution: academic = 16 (88.9%) vs. non−academic = 2 (11.1%). **(b)** Trial-phase distribution: early-phase trials predominate (Phase I = 4; I/II = 2; II = 2), with only one study each in Phase II/III and Phase III. Eight studies were labeled “Not Applicable” owing to observational, device feasibility, or registry designs lacking formal phase reporting.

To further delineate the organizational and developmental structure of MPLC-focused trials, we analyzed the distribution of study sponsors and clinical phases. Academic institutions were the primary sponsors in most cases, reflecting limited engagement from pharmaceutical industry and governmental agencies (**Figure 2a**). In terms of trial maturity, early-phase trials predominated, and no Phase IV study was identified. A substantial proportion of trials lacked a formal phase designation and were categorized as “Not Applicable,” which may indicate either non-pharmacological interventions (e.g., surgery or cryoablation), retrospective designs, or underreporting during registry submission (**Figure 2b**). This distribution underscores a critical gap in coordinated, later-phase efforts aimed at validating MPLC-specific treatment strategies.

One critical barrier to advancing MPLC research is the continued reliance on conventional staging systems—such as TNM classification (2)—which are inherently constructed for single-lesion evaluation. This staging logic fails to accommodate anatomically distinct, clonally independent lesions and further complicates enrolment in trials using RECIST-defined mono-lesion endpoints. As a result, MPLC patients are structurally excluded from trial frameworks that were never designed to reflect their disease reality.

## Root causes of structural exclusion

Our registry-based analysis confirms a stark reality: despite its growing recognition as a distinct clinical entity, MPLC is profoundly and systematically excluded from the modern clinical trial landscape. Only 0.22% of lung cancer studies explicitly include MPLC—this is not a simple oversight but a symptom of a deeper misalignment between conventional single-lesion trial design and the biological complexity of multifocal disease.

### The legacy of single-lesion frameworks and the RECIST barrier

The primary barrier to MPLC inclusion is the historical reliance on trial paradigms designed for a single, primary tumor (14). Modern oncology drug development is built upon this “one-tumor” model, with evaluation criteria that are fundamentally ill-suited for multifocal disease (15, 16). The most significant of these is the Response Evaluation Criteria in Solid Tumors (RECIST 1.1), a methodology that defines treatment response based on the dimensional changes in a limited number of “target lesions,” creating an immediate structural hurdle (14).

In an MPLC patient, who may harbor multiple, clonally distinct tumors, this approach is inherently flawed. It is biologically plausible that one lesion may respond to therapy while another, harboring a different molecular profile, remains stable or progresses. Under RECIST, such a divergent response could be averaged into a classification of “stable disease” or, worse, “progressive disease,” masking clinically meaningful benefits in specific lesions and potentially leading to the premature discontinuation of effective therapies. This rigid, mono-lesion-centric logic structurally disqualifies MPLC patients, whose disease reality cannot be captured by a single response metric.

### Biological heterogeneity and the challenge to precision oncology

The exclusion of MPLC is particularly problematic in the era of precision oncology. TRACERx and other multi-omics studies reveal marked inter- and intra-tumor heterogeneity even within a single lung lesion (17); MPLC magnifies this complexity across several anatomically distinct primaries. Each lesion can represent an independent evolutionary trajectory with a unique set of driver mutations, resistance mechanisms, and tumor microenvironments.

The existing evidence base, still dominated by academic, surgery-oriented observational series, highlights a persistent challenge in addressing this biological heterogeneity (18). Consequently, trials that evaluate a single-biomarker targeted therapy—such as an EGFR inhibitor—are intrinsically misaligned with the MPLC population. A meaningful evidence base will require designs that capture lesion-level molecular data and analyze differential responses, rather than applying a one-size-fits-all metric.

### Economic and logistical disincentives for industry sponsors

Nearly all MPLC studies are academically sponsored—evidence of major economic and logistical disincentives for the pharmaceutical industry. Commercially, MPLC constitutes a small, highly fragmented niche within the lung cancer market. Trial design is also more demanding: lesion-specific imaging, serial biopsies for molecular profiling, and complex statistical plans to accommodate composite end-points and divergent responses all drive up cost and complexity. Coupled with a higher failure risk from pronounced tumor heterogeneity, these factors make MPLC trials a poor fit for sponsors seeking reliable returns. This economic calculus—rarely acknowledged outright—remains a central reason for MPLC’s persistent invisibility in interventional research.

## Toward inclusion-oriented trial design

To bridge the gap between research frameworks and clinical reality, we recommend that future lung cancer trials incorporate inclusive design principles tailored to the biological and clinical complexity of MPLC. These may include stratified eligibility criteria that recognize multifocal lesions as independent entities, the use of ctDNA or lesion-level imaging to support composite endpoints, and the adaptation of response evaluation beyond RECIST-defined (19) single-lesion metrics. Surgical cohorts could benefit from integrating spatial transcriptomic or clonal evolution models, offering insight into lesion-level progression risk.

More broadly, guidelines for MPLC-specific trial design should be developed through multidisciplinary collaboration involving thoracic oncologists, pathologists, trial methodologists, and regulatory agencies. Recognizing MPLC not as a confounder but as a model of spatial–temporal tumor evolution is essential for advancing both trial inclusivity and precision oncology at large.

## Supplementary material

Supplementary Figure 1 PRISMA flow diagram: study selection process. This flowchart illustrates the screening and selection of interventional lung−cancer trials registered between January 2015 and May 2024 across ClinicalTrials.gov, ChiCTR, EU−CTR, and ISRCTN. At each stage, the number of records is indicated: records identified (n = 10 724), duplicates removed (n = 2 512), records screened (n = 8 212), records excluded (n = 7 803), full-text records assessed (n = 409), reports excluded with reasons (n = 391), and studies included in the review (n = 18). Reasons for full-text exclusion: not MPLC (n = 250); non−interventional (n = 80); withdrawn/terminated (n = 40); duplicate/incomplete records (n = 21).
